# Ultra-Long-Distance Hybrid BOTDA/Ф-OTDR

**DOI:** 10.3390/s18040976

**Published:** 2018-03-25

**Authors:** Yun Fu, Zinan Wang, Richeng Zhu, Naitian Xue, Jialin Jiang, Chongyu Lu, Bin Zhang, Le Yang, David Atubga, Yunjiang Rao

**Affiliations:** 1 Key Laboratory of Optical Fiber Sensing and Communications (Ministry of Education), University of Electronic Science and Technology of China (UESTC), Chengdu 611731, China; y_fu_uestc@163.com (Y.F.); 15520778390@163.com (R.Z.); naitian9325@163.com (N.X.); jryy_jiang@163.com (J.J.); titanlcy@163.com (C.L.); ligongzhangbin@163.com (B.Z.); evoenzo@163.com (L.Y.); atubgadavid@ymail.com (D.A.); 2 Center for Information Geoscience, UESTC, Chengdu 611731, China

**Keywords:** fiber optics sensors, optical time domain reflectometry, Rayleigh scattering, stimulated Brillouin scattering, optical amplifiers

## Abstract

In the distributed optical fiber sensing (DOFS) domain, simultaneous measurement of vibration and temperature/strain based on Rayleigh scattering and Brillouin scattering in fiber could have wide applications. However, there are certain challenges for the case of ultra-long sensing range, including the interplay of different scattering mechanisms, the interaction of two types of sensing signals, and the competition of pump power. In this paper, a hybrid DOFS system, which can simultaneously measure temperature/strain and vibration over 150 km, is elaborately designed via integrating the Brillouin optical time-domain analyzer (BOTDA) and phase-sensitive optical time-domain reflectometry (Ф-OTDR). Distributed Raman and Brillouin amplifications, frequency division multiplexing (FDM), wavelength division multiplexing (WDM), and time division multiplexing (TDM) are delicately fused to accommodate ultra-long-distance BOTDA and Ф-OTDR. Consequently, the sensing range of the hybrid system is 150.62 km, and the spatial resolution of BOTDA and Ф-OTDR are 9 m and 30 m, respectively. The measurement uncertainty of the BOTDA is ± 0.82 MHz. To the best of our knowledge, this is the first time that such hybrid DOFS is realized with a hundred-kilometer length scale.

## 1. Introduction

Distributed optical fiber sensing (DOFS) systems play indispensable roles in many fields, such as oil and gas industries, geophysical sciences, and structural health monitoring [1]. The sensing range of DOFS is an important parameter since it reflects the ultimate capability of a system [2,3]. The essence of range extension is the signal-to-noise ratio (SNR) enhancement, so that numerous methods published can be used for the purpose [2]. Combined with the techniques of distributed Raman amplification (DRA) [4], random fiber lasing amplification (RFLA) [5,6], and Simplex coding [7,8], the sensing range of BOTDA has been extended to 154.4 km [6] and then 157.47 km [9] with non-local means (NLM) filtering [10]. On the other hand, if half of the fiber is used for sensing, and the other half is used for transmitting the probe light [11,12], the whole fiber length of BOTDA has achieved 240 km [13]. For Ф-OTDR, the sensing distance has reached 175 km [14] when the DRA, RFLA, distributed Brillouin amplification (DBA) [15], and coherent detection [16,17,18] are all used.

In many applications, both static and dynamic sensing capabilities are required [19,20]. For example, the temperature and intrusion monitoring are both important for pipeline safety; the information of both strain and vibration is needed for large-scale structures, such as bridges and tunnels [1]. However, due to the complex interplay among scattering effects inside fiber, as well as the competition of the pump power, it has never been reported that such hybrid DOFS could be realized with a hundred-kilometer length scale.

In this paper, we integrate the sensing mechanisms based on stimulated Brillouin scattering (SBS) and Rayleigh scattering (RS), for ultra-long-distance static (temperature/strain) and dynamic (vibration) measurements at the same time. The difficulties to be overcome include the interplay of different scattering mechanisms, the interaction of two sensing signals, and the competition of pump power. Therefore, the techniques of distributed amplification, FDM, WDM, and TDM are delicately fused in the system. In detail, BOTDA and Ф-OTDR share the distributed gains of the Raman pump (RP) and random fiber laser (RFL). The FDM technology is used to make the Brillouin pump provide gain only in the specific segment, where use of the RP and RFL cannot sufficiently amplify the RS light; meanwhile, it can also avoid the non-local effects of BOTDA. WDM and the interleaving of input pulses (TDM) are both employed to minimize the interaction between BOTDA and Ф-OTDR. As a result, the achieved sensing range of the hybrid system is 150.62 km and the spatial resolution of BOTDA/Ф-OTDR is 9 m/30 m. The measurement uncertainty of BOTDA is ± 0.82 MHz. To the best of our knowledge, it is the first time that the sensing range of such an integrated system achieves over a hundred-kilometer length scale.

## 2. Operation Principles

There are certain difficulties that need to be overcome when the hybrid ultra-long DOFS system is designed. Firstly, in order to extend the sensing range, the DRA and RFLA techniques are used in the system [6]. However, if the pump power is not adjusted properly, even though the hybrid gains of the RP and RFL can meet the requirement of BOTDA, the RS signals cannot be measured from the middle of the fiber link [14]. Hence, FDM is applied to make the Brillouin pump provide gains in the specific segment. Also, in order to separate the signals of BOTDA and Ф-OTDR, WDM is utilized. In addition, the overlap of two types of input pulses results in the cross phase modulation (XPM), four-wave mixing (FWM), and the competition of the pump power, so the interleaving of probe pulses is arranged, i.e., TDM.

**Figure 1:**
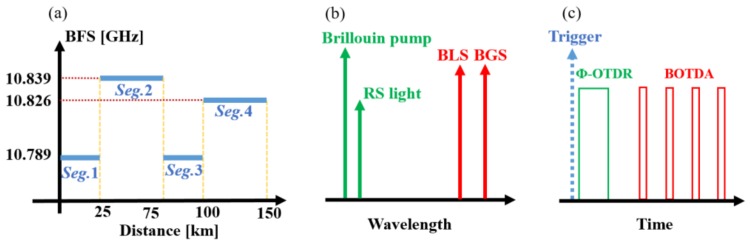
Diagram of operation principle. (**a**) Arrangement of the fiber segments had different Brillouin frequency shifts (BFSs); (**b**) wavelengths used in BOTDA and Ф-OTDR. RS: Rayleigh scattering; BLS: Brillouin loss spectrum; BGS: Brillouin gain spectrum; (**c**) interleaving of the input pulses of BOTDA and Ф-OTDR.

The hybrid of DRA, RFLA, and DBA is the key technique for sensing range extension. For BOTDA, the hybrid gains of the RP and RFL can make the sensing range beyond 150 km [6]. On the other hand, under the same amplifying condition, the RS light drops to a low level around 120 km, so the Brillouin pump is used to enhance the SNR in the specific section [14]. To make the best use of the Brillouin pump, FDM is utilized in this system, as shown in Figure 1a [21]. The frequency differences of BFSs between the neighboring segments are larger than 30 MHz due to the 3 dB bandwidth of the Brillouin gain spectrum (BGS) [22]. Hence, the employment of FDM can make the Brillouin pump generate significant gain only in Segment 3 (Seg. 3) [14]. FDM allows the signal power to maintain a proper level along the fiber, and economizes the Brillouin pump power until it transmits to the specific location [15]. Meanwhile, FDM can be used to suppress the non-local effects efficiently [21].

When BOTDA and Ф-OTDR operate simultaneously, it is important to separate the signals of the two subsystems. Hence, WDM is used, as shown in Figure 1b. The center wavelength of BOTDA is the average of BLS and BGS; the center wavelength of Ф-OTDR is in agreement with the RS light. The difference of the two center wavelengths should be large enough that the dense wavelength division multiplexer (DWDM) can filter out each waveband, while the difference should be small enough to ensure that the center wavelengths of BOTDA and Ф-OTDR are well located within the amplifying wavebands of both the RP and RFL.

In addition, without pulse-interval control of BOTDA and Ф-OTDR, their pulses could be overlapped. This leads to XPM, FWM, and the competition of the pump power. In order to avoid these harmful effects, interleaving between the pulses of Ф-OTDR and BOTDA is designed, as shown in Figure 1c, while the two subsystems share the same triggers and have the same repeating periods. In Figure 1c, the input pulses of BOTDA show the first column of Simplex coding.

**Figure 2:**
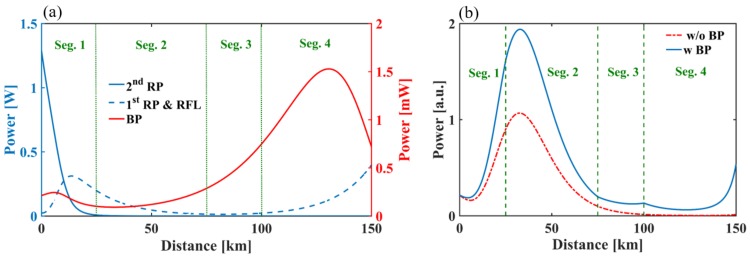
Simulation results of distributed amplification. RP: Raman pump; BP: Brillouin pump; RFL: random fiber laser. (**a**) Pump power distribution along the fiber; (**b**) Rayleigh backscattering traces with/without Brillouin pump.

To optimize the power of each pump, a simulation is carried out and the results are shown in Figure 2. For BOTDA, the power of the probe light is set at −12.56 dBm, while the peak power of the pulses is set at 13.57 dBm. In the case of Ф-OTDR, the peak power of input pulses is set at 13.97 dBm. Figure 2a shows the pump power distribution along the fiber, in the case that the input power of first/second order Raman pump is 26.5/30.8 dBm. It should be noted that the power of the second order Raman pump should not exceed 33.4 dBm when the power of the first order Raman pump is 26.5 dBm, otherwise the random fiber laser of 1550 nm would be produced. According to Figure 2a, it can be seen that the hybrid power of the RP and RFL achieves relatively high levels in Seg. 1 and Seg. 4, which can extend the sensing range of BOTDA beyond 150 km [6,9]. However, without the Brillouin pump, the hybrid gain is relatively low in Seg. 3, which cannot meet the amplification requirement of RS light, as shown in the red dashed line in Figure 2b. Hence, the Brillouin pump is designed to only amplify within Seg. 3, and its power distribution and effects are shown as the red line in Figure 2a and the blue solid line in Figure 2b, respectively. The input power of the Brillouin pump is −1.43 dBm, and it increases to −0.84 dBm at the position of 100 km due to the first order Raman pump. According to Figure 2b, without the Brillouin pump, the power of the received light at 100 km is only 1% of the peak value which is located at ~35 km; however, with the Brillouin pump, an obvious improvement of the signal power can be seen in Seg. 3 and Seg. 4.

## 3. Experimental Setup

**Figure 3:**
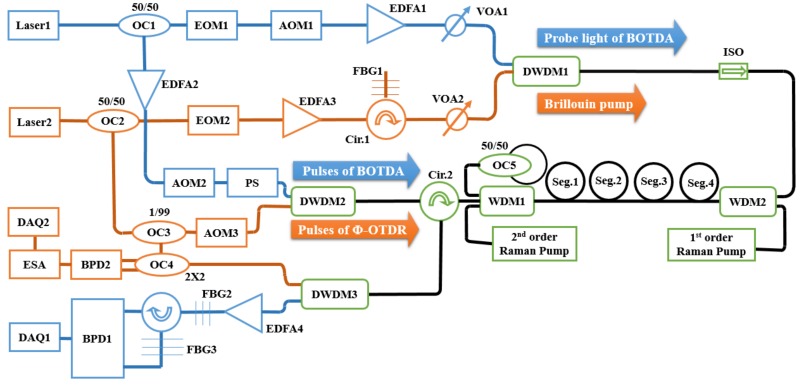
Experimental setup. OC: optical coupler; EOM: electric-optical modulator; AOM: acoustic optical modulator; EDFA: erbium-doped fiber amplifier; VOA: adjustable attenuator; ISO: isolator; FBG: fiber Bragg grating; PS: polarization scrambler; WDM: wavelength division multiplexer; DWDM: dense wavelength division multiplexer; BPD: balanced photo-detector; DAQ: data acquisition.

The experimental setup of the ultra-long DOFS system is shown in Figure 3. The blue section shows the BOTDA, while the orange section shows the Ф-OTDR, and the green section with black lines represents the shared component.

With respect to BOTDA, the dual-sideband of probe light is used to decrease the non-local effect [23], and balanced detection is used to enhance the SNR [12,24]. In detail, the probe light is modulated by EOM1, and AOM1 is used to make the center frequencies of probe light and pulses equal. FBG2 is used to remove the carrier light, while FBG3 is used to separate the BGS and BLS, so balanced detection can be used effectively in the system [13]. In addition, 127-bit Simplex coding is used to enhance the received SNR [7], and the final sensing results are obtained after averaging 16 times. Polarization scrambler (PS) in Figure 3 is used to minimize the effect of polarization [25].

In the case of Ф-OTDR, coherent detection and envelope demodulation are utilized. The light that is output from the 1% port of OC3 is the local oscillator (LO). OC4 mixes the LO and the RS light, and balanced detection is used after OC4 [14]. The electrical spectrum analyzer (ESA) is adjusted to realize envelope demodulation before the Rayleigh signals input into DAQ2 [14]. The vibration signals are solved after averaging 10 times.

The parameters of the experimental setup are shown in the following. The input pump power set in the experiment is consistent with the simulation parameters shown in Section II. Besides, for all the DWDMs, the center frequency differences between the two channels are 100 GHz, and the FWHM of each channel is 0.65 nm. The frequency shifts of AOM1 and AOM2 are 200 MHz, and the frequency shift of AOM3 is 80 MHz. The bandwidth of each balanced photo-detector (BPD) is 350 MHz. The pulse duration of BOTDA/Ф-OTDR is 90/300 ns. The time interval between the falling edge of the pulse used in Ф-OTDR and the rising edge of the first pulse in BOTDA is 500 ns. The repeating periods of BOTDA and Ф-OTDR are both 1.6 ms. The sampling rate of BOTDA is 100 MHz, while the sampling rate of Ф-OTDR is 25 MHz. In addition, a roll of fiber whose length is about 1 km is added at the start of the four segments to extend the sensing range.

## 4. Results and Discussion

**Figure 4:**
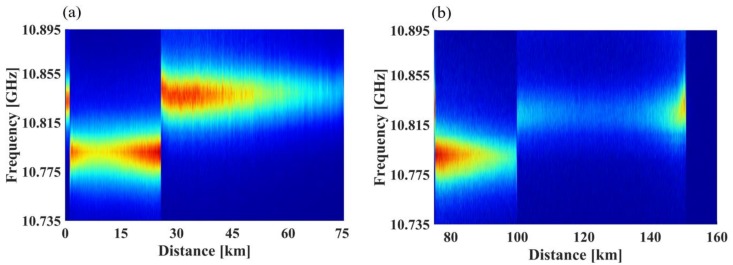
BGS as the function of distance. (**a**) BGS in the range from 0 to 75 km; (**b**) BGS in the range from 75 km to the end of the fiber.

**Figure 5:**
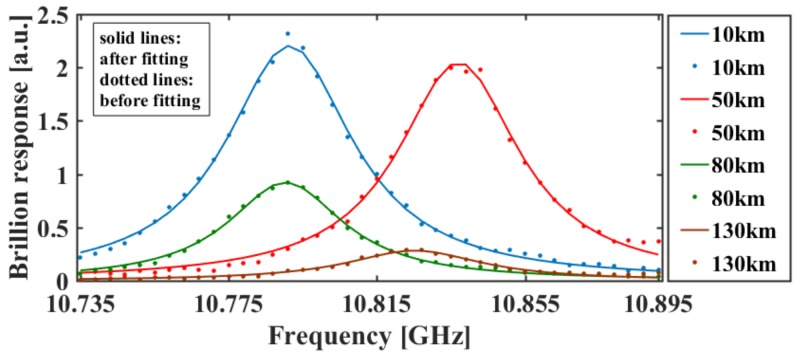
BGSs at different positions.

Figure 4 shows the BGS of the fiber under test (FUT) when BOTDA and Ф-OTDR operate simultaneously. The frequency sweep range is from 10.735 GHz to 10.895 GHz, while the sweep interval is 4 MHz. Figure 5 shows some vertical sections of Figure 4. It is lucid that the experimental data are significantly consistent with the fitting Lorentzian curves, and there is no obvious appearance of dual peaks and asymmetry of BGS. Hence, the SNR maintains a high level along the FUT, and the non-local effect does not appear in all the segments [26].

**Figure 6:**
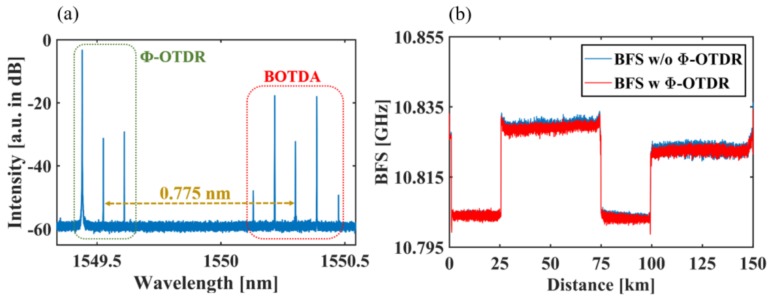
Demonstration that Ф-OTDR has little influence on the final sensing results of BOTDA. (**a**) Optical spectrum measured before DWDM3; (**b**) Brillouin frequency shift (BFS) with/without the Ф-OTDR.

When BOTDA and Ф-OTDR operate simultaneously, the impact between the two subsystems should be evaluated. Figure 6a shows the optical spectrum measured before DWDM3. The wavelength difference between the two lasers is 0.775 nm, so that the signals of BOTDA and Ф-OTDR can be well separated by DWDM3. Figure 6b shows the BFS with/without the operation of Ф-OTDR. The standard deviation of the difference between the two traces is only 0.047 MHz, which indicates that Ф-OTDR has little impact on the sensing results of BOTDA.

**Figure 7:**
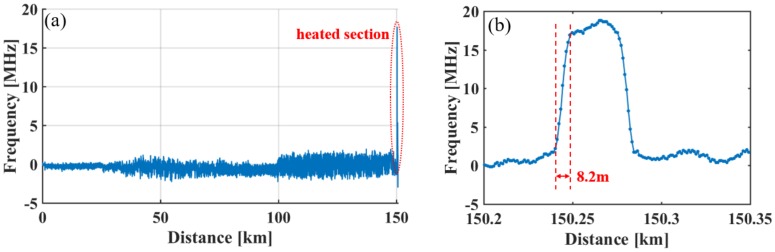
BFS variation versus distance: (**a**) along the whole sensing fiber; (**b**) around the heated section.

When the two subsystems operate simultaneously, the accuracy of BOTDA is measured. An approximately 35 m long fiber located near the terminal (150.23 km) is heated by a temperature controlled chamber. The applied temperature difference at the heated section is 18.2 °C. Figure 7a shows the BFS variance along the whole FUT. It can be seen that the noisiest section locates in Seg. 4, where the uncertainty is ±0.82 MHz. Figure 7b demonstrates the enlarged view of the heated section shown in Figure 7a. It can be seen that the measured spatial resolution is 8.2 m. The measured temperature difference is 18.7 °C, which is consistent with the applied change of temperature. Therefore, the figure of merit (FoM) of BOTDA [27] is above 35,000. Compared with [19,20], the system shown in the paper has a longer sensing range and better performance.

**Figure 8:**
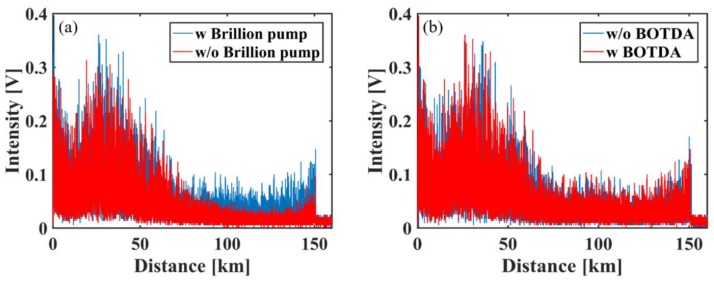
Experiment results of Ф-OTDR. (**a**) Ф-OTDR intensity traces with the operation of BOTDA; (**b**) Ф-OTDR intensity traces with the Brillouin pump.

**Figure 9:**
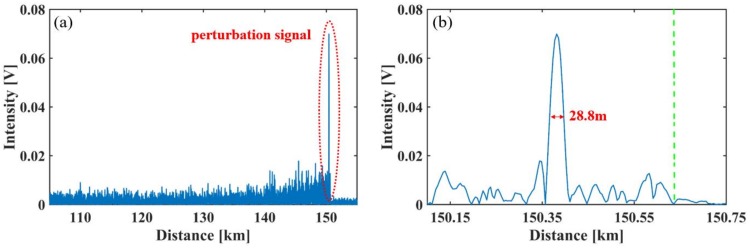
Demodulated intrusion signals at the position of 150.37 km. (**a**) Along the fiber; (**b**) around the perturbed spot.

Then, the results of Ф-OTDR are presented in Figure 8 and Figure 9. Figure 8a shows the RS traces with BOTDA and with/without the Brillouin pump. It can be seen that the Brillouin pump enhances the signal power significantly in Seg. 3, which is consistent with the simulation results shown in Figure 2b. After the amplification of Seg. 3, the remainder of the Brillouin pump also improves the SNR of Seg. 1 a little bit. Hence, the power of scattering trace exhibits overall improvement compared to the case without the Brillouin pump. Figure 8b shows the scattering signals with the Brillouin pump and with/without the operation of BOTDA, and it can be seen that BOTDA has little detrimental impact on the profile of Ф-OTDR traces. In a word, the simultaneous operation of BOTDA and Ф-OTDR is viable.

The external vibration signals can be detected through the intensity difference between Rayleigh backscattering traces [14]. Figure 9 shows the demodulated perturbation signal at the position of 150.37 km. It should be noted that without the Brillouin pump, the vibration cannot be solved. Figure 9a shows the Ф-OTDR trace difference versus distance. Figure 9b shows the details of the perturbation signal shown in Figure 9a. The calculated SNR of the vibration signal is 15.14 dB, and the sensing range is 150.62 km, which is marked by the green dashed line. The measured spatial resolution is 28.8 m.

## 5. Conclusions

In this paper, we report an ultra-long DOFS system, which can measure static and dynamic events at the same time, by fusing BOTDA and Ф-OTDR that share the same fiber link. The difficulties related to the competition of amplifying pumps and the interaction between two types of signals are properly overcome. The key techniques applied in the system include hybrid distributed amplifications, FDM, WDM, and TDM. As a result, the achieved sensing range is 150.62 km. The experimental results prove that the operation of one subsystem has little detrimental impact on the performance of the other subsystem. The spatial resolutions of BOTDA and Ф-OTDR are 9 m and 30 m, respectively, and the measurement uncertainty of BOTDA is ± 0.82 MHz. It is the first time that such an integrated system is realized with a hundred-kilometer length scale, to the best of our knowledge.
